# A Unique Profile of Adenomatous Polyposis Coli Gene Mutations in Iranian Patients Suffering Sporadic Colorectal Cancer

**Published:** 2014-02-03

**Authors:** Mojtaba Hasanpour, Hamid Galehdari, Abdolrahim Masjedizadeh, Naser Ajami

**Affiliations:** 1 Toxicology Research Center, Ahvaz Jundishapur University of Medical Sciences, Ahvaz, Iran; 2 Department of Genetics, Faculty of Science, Shahid Chamran University, Ahvaz, Iran; 3 Department of Gastrology, Ahvaz Jundishapur University of Medical Sciences, Ahvaz, Iran

**Keywords:** Colorectal Cancer, *APC*, Iran

## Abstract

**Objective:**

Colorectal cancer (CRC) is one of the most common and aggressive cancers worldwide. The majority of CRC cases are sporadic that caused by somatic mutations. The Adenomatous Polyposis Coli (*APC*; OMIM 611731) is a tumor suppressor gene of Wnt pathway and is frequently mutated in CRC cases. This study was designed to investigate the spectrum of *APC* gene mutations in Iranian patients with sporadic colorectal cancer.

**Materials and Methods:**

In this descriptive study, Tumor and normal tissue samples were obtained from thirty randomly selected and unrelated sporadic CRC patients. We examined the hotspot region of the *APC* gene in all patients. Our mutation detection method was direct DNA sequencing.

**Results:**

We found a total of 8 different *APC* mutations, including two nonsense mutations (c.4099C>T and c.4348C>T), two missense mutations (c.3236C>G and c.3527C>T) and four frame shift mutations (c.2804dupA, c.4317delT, c.4464_4471delATTACATT and c.4468_4469dupCA). The c.3236C>G and c.4468_4469dupCA are novel mutations. The overall frequency of *APC* mutation was 26.7% (8 of 30 patients).

**Conclusion:**

This mutation rate is lower in comparison with previous studies from other countries. The findings of present study demonstrate a different *APC* mutation spectrum in CRC patients of Iranian origin compared with other populations.

## Introduction

Colorectal cancer (CRC) is the third most common cancer in men and the second in women worldwide. The incidence rates of CRC in Iranian males and females are 8.7 and 6.4 in 100,000 respectively (1). Compared with Western countries, CRC incidence rates are low in south Asia, but recent studies in Iran have shown a significant increase in the rate of colorectal cancer (2-4). The majority (75-80%) of CRC cases is without a family history and arises by somatic mutations in colon and rectum (5, 6). Mutations in the *APC* gene occur in 34- 80% of sporadic CRC cases (7, 8). The *APC* tumor suppressor gene consists of 15 exons with exon 15 covering more than 75% of the coding sequence. About 60% of all mutations in *APC* occur in the mutation cluster region (MCR) between codons 1286 and 1513 in exon 15 (9, 10). The majority of *APC* mutations in the MCR introduce a stop codon, resulting in a truncated protein that lacks the binding site for two important interactants, β-catenin and axin, which act together in the Wnt signaling pathway (11).

The *APC* protein participates in many of the fundamental cellular processes such as proliferation, differentiation, migration and apoptosis (12). This protein is a negative regulator of the Wnt signaling pathway and is involved in cellular proliferation and differentiation. *APC* is also involved in the dynamics of cytoskeleton, and has an impact on apoptosis (13-15). This multifunctionality of *APC* protein may explain why disrupting *APC* is harmful to the epithelium of the intestine (12).

In 1990, Fearon and Vogelstein (16) first proposed a multistep genetic model for colorectal tumorigenesis. According to this hypothesis, activation of the Wnt signaling through disruption of *APC* gene is the earliest genetic event in colorectal tumorigenesis. Inactivation of *APC* gene occurs by mutation, loss of heterozygosity (LOH) or promoter hypermethylation (9). Due to the lack of a systematic investigation of *APC* gene mutations in Iran, we attempted to screen the MCR region for putative changes in a cohort of Iranian individuals that suffer from CRC.

## Materials and Methods

### Patients

In this descriptive study, fresh colon tissue from thirty sporadic CRC patients with no family history (18 men, 12 women) were collected that referred to Mehr and Emam hospitals in Ahvaz in the period from November 2009 to February 2011. Tumor and adjacent normal tissue specimens were obtained from all patients after obtaining formal consent and were stored at -80˚C until use. The specimens had the histopathologic characteristics of adenocarcinoma.

This project was approved by the Ethical Committee of Ahvaz Jundishapur University of Medical Sciences.

### Genomic DNA extraction

The DNA from tumor and normal tissue was separately extracted using the AccuPrep® Genomic DNA Extraction Kit (Bioneer Corporation, Daejeon, Korea).

### Primers and polymerase chain reaction

**Fig 1:**
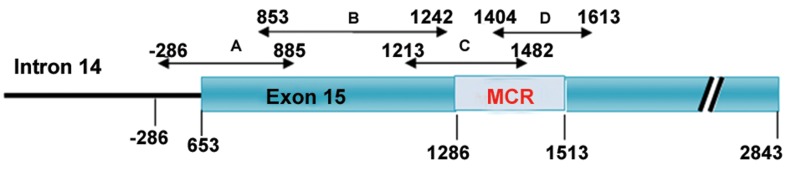
The structure of the *APC* gene is partly demonstrating the intron 14 and exon 15. Horizontal arrows show the positions of the overlapped fragments covering the nucleotide -286 in intron 14 and codon 1613 within exon 15.

Four primer pairs were designed for four overlapping fragments (codons 653-885, 853- 1242, 1213-1482 and 1404-1613) of exon 15 (Table 1). The positions and the size of polymerase chain reaction (PCR) products are illustrated in figure 1. Amplification was performed in a total volume of 30 μl of reaction mixture containing 80-130 ng genomic DNA, 1X PCR buffer, 1.5 mmol/L of MgCl2, 0.2 mmol/L of dNTP, 0.2-0.4 μmol/L of each primer, and 3 U Super Taq DNA polymerase (Gen Fanavaran Ldt, Tehran, Iran). PCR conditions were as follows: initial denaturation at 94˚C for 5 minutes, 30 cycles of denaturation at 94˚C for 1 minute, annealing for 45 seconds and extension at 72˚C for 1 minute, and a final extension at 72˚C for 7 minutes.

**Table 1:** Primers characteristics using for PCR amplification.


Fragment	Primer	Ta (°C)	Fragment length (bp)

**A**	AF: 5´-AGTAAATGTATGTGCCCCACCCCC-3´	68	984
	AR: 5´-GGGCTGCAGTGGTGGAGATCTG-3´		
**B**	BF: 5´-TGGAGAGAGAACGCGGAATTGG-3´	66	1173
	BR: 5´-GCTGACCACTTCTACTCTGTGCAG-3´		
**C**	CF: 5´-CAAGCAGTGAGAATACGTCCACAC-3´	64	808
	CR: 5´-AGAACCTGGACCCTCTGAACTGCA-3´		
**D**	DF: 5´-TCCGTTCAGAGTGAACCATGCA-3´	65	628
	DR: 5´-GCAGCTGACTTGGTTTCCTTGCCA-3´		


### DNA sequencing

The purified PCR products were sequenced by Macrogen (Seoul, Korea) using an Applied Biosystems 3730 DNA Analyzer. Sequence chromatograms were analysed using the Chromas software and NCBI BLAST tool. When a mutation was found in a tumor DNA sample, the corresponding normal DNA was systematically checked for the absence of this mutation.

## Results

**Fig 2:**
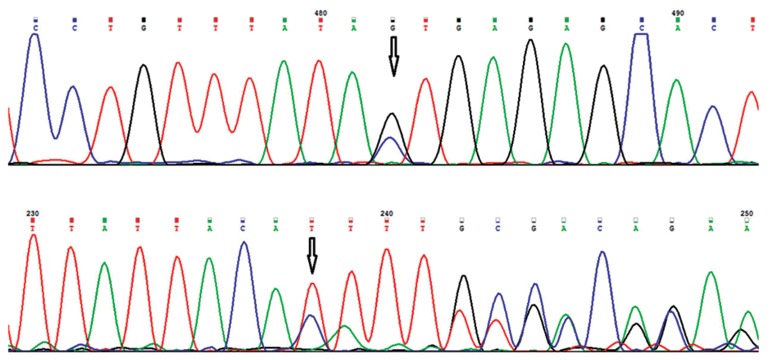
DNA sequences of novel *APC* mutations. A. Chromatogram of c.3236C>G (p.Thr1079Ser) mutation. B. Chromatogram of c.4468_4469dupCA (p.Phe1491IlefsX17) mutation.

Genomic DNA sequencing of codon 653 to 1613 of *APC* gene and exon 15-intron 14 boundary enabled us to identify a total of 8 different mutations in 8 sporadic CRC patients (Table 2), including two nonsense mutations (25%), two missense mutations (25%) and four frameshift mutations (50%). Two of 8 mutations namely c.3236C>G (p.Thr1079Ser) and c.4468_4469dupCA (p.Phe1491IlefsX17) have not previously been reported in Human Gene Mutation Database (HGMD), Sanger-*APC* mutations database, the Leiden Open Variation Database (LOVD) and the literature, indicating that they are novel mutations (Table 3, Fig 2). The c.3236C>G mutation (Fig 2A) results in an amino acid substitution (p.Thr1079Ser) with unknown effect and the c.4468_4469dupCA mutation (Fig 2B) leads to a premature stop-codon at position 1507. The remaining 6 *APC* mutations had been reported previously. Of the 8 identified mutations, 5 (62.5%) occurred in the MCR. All mutations, with the exception of p.Thr1079Ser and p.Pro1176Leu, lead to truncated *APC* protein. The patients 7 and 18 showed two mutations; each had a truncating mutation and a missense change. The overall frequency of *APC* mutation was 26.7% (8 of 30 cases). Pathogenic mutation was found in 7 patients (23.3%) of which 6 (20% of total) showed mutation in the MCR. We detected three polymorphisms, including one non-synonymous (p.Glu1317Gln), one synonymous (p.Thr1493Thr) and one intronic (c.1959-143_1959-140dupAGAA) polymorphism. These polymorphisms were reported previously according to Human Variation database. Two patients had p.Glu1317Gln polymorphism. This non-synonymous variant is considered to play a role in the development of colorectal tumors (17). The p.Thr1493Thr polymorphism was observed in twenty three patients. Twenty two patients carrying the c.1959-143_1959-140dupAGAA polymorphism. We also found the novel variant c.3345G>T (p.Val1115Val) in 2 patients. Human Variation: http://www.ncbi.nlm.nih.gov/projects/SNP/ tranSNP/tranSNP.cgi.

**Table 2:** *APC* mutation status in relation to clinicopathological variables.


Characteristic	Frequency (%)	Yes (n=8)	No (n=22)

**Age at diagnosis**			
<60	12 (40)	4 (33.3%)	8 (66.7%)
≥60	18 (60)	4 (22.2%)	14 (77.8%)
**Sex**			
Male	18 (60)	4 (22.2%)	14 (77.8%)
Female	12 (40)	4 (33.3%)	8 (66.7%)
**Tumor location**			
Right colon	12 (40)	2 (16.7%)	10 (83.3%)
Left colon	9 (30)	3 (33.3%)	6 (66.7%)
Rectum	9 (30)	3 (33.3%)	6 (66.7%)
**Tumor histology**			
Poorly differentiated	2 (6.67)	0	2 (100%)
Moderately differentiated	11 (36.67)	4 (36.4%)	7 (63.6%)
Well differentiated	17 (56.67)	4 (23.5%)	13 (76.5%)


**Table 3:** *APC* mutations in Iranian sporadic CRC patients; novel mutations are in bold.


Patient ID	DNA change	Protein change	Mutation type	Origin

**3 & 7 **	c.3527C>T	p.Pro1176Leu	Missense	Somatic
**7**	c.2804dupA	p.Tyr935fsX1	Frameshift	Somatic
**18 **	**c.3236C>G**	**p.Thr1079Ser**	Missense	Germline
**18**	c.4099C>T	p.Gln1367X	Nonsense	Somatic
**20**	c.4317delT	p.Pro1440HisfsX33	Frameshift	Somatic
**23 & 30**	c.4348C>T	p.Arg1450X	Nonsense	Somatic
**25**	c.4464_4471delATTACATT	p.Leu1488PhefsX23	Frameshift	Somatic
**28**	**c.4468_4469dupCA**	**p.Phe1491IlefsX17**	Frameshift	Somatic


## Discussion

The CRC has a high incidence of mortality in western countries that was also subjected for intensive investigations. Otherwise, its molecular characteristics remained widely unknown in Middle Eastern countries. Recent epidemiologic studies in Iran show rapid increase in the rate of CRC (2, 4) and with rising of the CRC cases in the developing countries, attention should be given to extend the underlying molecular knowledge of this mostly heterogeneous cancer (18). For instance, beside (loss of heterozygosity) LOH and hypermethylation, inactivation of the *APC* gene by mutation has been observed in different type of colon cancer, such as familial or sporadic and with or without polyposis (9). It is widely accepted that the *APC* gene inactivation is the first event in a multistep process of the CRC (16). Surprisingly and because of heterogeneous nature of the sporadic CRC, some tumors show no mutation in the *APC* gene and some others reveal high mutation rate (19). This attitude appears to be dependent on ethnicity, geographic region, dietary and genetic predisposition (20, 21).

In the present study, the *APC* gene was subjected for mutation survey in individuals suffering from sporadic type of CRC in southwest Iran.

We observed a mutation rate of 26.7% in a large part of exon 15 in the *APC* gene. Truncating mutations (nonsense and frameshift mutations) in the MCR were observed in 6 (20%) tumor samples, which is in concordance with the rate in Hungary and Tunisia (22, 23), but lower than in other European countries, the USA and Japan (Table 4, 24- 29). The mutation cluster region (codon 1286 to 1513) corresponds to the 20-amino acid sequence, exhibiting the β-catenin and the axin binding sites (9). A truncating mutation in the MCR abolishes this functional domain, which in turn leads to the cytoplasmic and nuclear accumulation of the β-catenin protein in the colorectal cells. The β-catenin accumulation is associated with activation of Wnt signaling pathway that promotes the generation of tumors (30).

Seven of our patients (23.3%) showed here somatic inactivation of one of the *APC* alleles. Besides mutation in other part of the *APC* gene, LOH and promoter hypermethylation may be the possible second hit mechanism in our cases. From two mutation hotspots at codons 1309 and 1450 (31), we only found 2 cases with nonsense mutation at codon 1450. The p.Phe1491IlefsX17 frameshift mutation and the p.Thr1079Ser missense mutation are novel that have not been published before. All detected nonsense and frameshift mutations in this study lead to the truncated form of the *APC* protein, underlining their pathogenic nature. The functional consequence of the p.Thr1079Ser and the p.Pro1176Leu changes is not clear. Furthermore, we propose these amino acid substitutions may cause damaging impact on the structure and function of *APC* protein. We also found a novel variant (p.Val1115Val) that its effect in the development of CRC is unknown and needs to be screened in a number of normal individuals.

**Table 4:** Frequency of *APC* mutation in different populations.


Country /Study	The studied area (codons)	Number of patients	Frequency of APC mutation (%)

Iran (present study)	653-1613	30	23.3
Hungary (22)	1285-1465	70	21.4
Tunisia (23)	1240-1513	48	20.8
Netherland (24)	1286-1520	656	37.3
Germany (25)	1260-1547	99	49.5
Norway(8)	653-2843	218	66
UK (11)	1028-1712	106	56.6
France(26)	653-2843	85	57.6
USA(27)	1286-1585	90	34.4
Japan(28)	582-1580	61	47.5
South Korea(29)	1202-1674	78	33.3


## Conclusion

Our findings demonstrate a unique *APC* mutation profile in Iranian CRC patients and support the idea that the spectrum of somatic *APC* mutations in CRCs are considerably variable and distinct among populations (32).

Although inactivating mutation of the *APC* gene was present in 23.3% of all the tumor cases studied, the actual percentage might be higher, because the present study focused only on the 5' half of *APC* exon 15, which includes the mutation cluster region. Hence, further mutation studies have to be conducted for the whole length of the *APC* gene for more evaluation. We also conclude that the MCR does not represent the hotspot region for mutation, at least in Iranian CRC patients.

To date, some hundred mutations have been reported in the *APC* gene. Some of these mutations occur across all ethnicity and populations and some others are specific to distinct geographic regions. The novel mutations presented in this study may be private to this region and therefore need to be screened country- wide in a large cohort of sporadic CRC patients in Iran.
